# Development and Characterization of an Oncolytic Human Adenovirus-Based Vector Co-Expressing the Adenovirus Death Protein and p14 Fusion-Associated Small Transmembrane Fusogenic Protein

**DOI:** 10.3390/ijms252212451

**Published:** 2024-11-20

**Authors:** Kathy L. Poulin, Ryan G. Clarkin, Joshua Del Papa, Robin J. Parks

**Affiliations:** 1 Regenerative Medicine Program, Ottawa Hospital Research Institute, Ottawa, ON K1H 8L6, Canada; 2 Department of Biochemistry, Microbiology, and Immunology, Faculty of Medicine, University of Ottawa, Ottawa, ON K1N 6N5, Canada; 3 Eric Poulin Centre for Neuromuscular Disease, University of Ottawa, Ottawa, ON K1N 6N5, Canada; 4 Department of Medicine, University of Ottawa, Ottawa, ON K1N 6N5, Canada

**Keywords:** adenovirus, oncolytic virus, transgene, adenovirus death protein, fusion-associated small transmembrane protein, FAST, self-cleaving peptide

## Abstract

Human adenovirus (HAdV)-based oncolytic vectors, which are designed to preferentially replicate in and kill cancer cells, have shown modest efficacy in human clinical trials in part due to poor viral distribution throughout the tumor mass. Previously, we showed that expression of the p14 fusion-associated small transmembrane (FAST) fusogenic protein could enhance oncolytic HAdV efficacy and reduce tumor growth rate in a human xenograft mouse model of cancer. We now explore whether co-expression of the adenovirus death protein (ADP) with p14 FAST protein could synergize to further enhance oncolytic vector efficacy. ADP is naturally encoded within the early region 3 (E3) of HAdV, a region which is frequently removed from HAdV-based vectors, and functions to enhance cell lysis and progeny release. We evaluated a variety of approaches to achieve optimal expression of the two proteins, the most efficient method being insertion of an expression cassette within the E3 deletion, consisting of the coding sequences for p14 FAST protein and ADP separated by a self-cleaving peptide derived from the porcine teschovirus-1 (P2A). However, the quantities of p14 FAST protein and ADP produced from this vector were reduced approximately 10-fold compared to a similar vector-expressing only p14 FAST protein and wildtype HAdV, respectively. Compared to our original oncolytic vector-expressing p14 FAST protein alone, reduced expression of p14 FAST protein and ADP from the P2A construct reduced cell-cell fusion, vector spread, and cell-killing activity in human A549 adenocarcinoma cells in culture. These studies show that a self-cleaving peptide can be used to express two different transgenes in an armed oncolytic HAdV vector, but also highlight the challenges in maintaining adequate transgene expression when modifying vector design.

## 1. Introduction

Cancer is a leading cause of morbidity and mortality worldwide, with millions of deaths occurring from the disease each year [1]. Despite advances in treatment, cancer prognoses remain grim and approximately 50% of all cancer patients will succumb to their disease, illustrating the need for novel and effective therapeutics. Oncolytic viruses comprise an emerging class of anticancer therapeutics that preferentially replicate in and kill cancer cells, but leave healthy tissue relatively unharmed [2,3]. Oncolytic or conditionally replicating adenoviruses (CRAds), in particular, have shown promising results in preclinical studies and demonstrated modest efficacy in human clinical trials [4,5]. However, poor viral spread throughout the tumor mass represents a major limitation of many oncolytic viruses, including CRAd [6,7,8]. Heterologous expression of fusogenic proteins, such as the gibbon-ape leukemia virus or measles virus fusion proteins, has been investigated as a means to increase viral spread throughout the tumor [9,10,11]. When expressed from oncolytic vectors, these fusogenic proteins cause infected cells to fuse with neighbouring cells, which effectively enhances spread of the effects of the virus and gene transfer throughout the tumor [12,13,14,15,16].

The p14 Fusion Associated Small Transmembrane (p14 FAST) protein is a small (14 kDa) fusogenic protein from a reptilian reovirus [17,18] that was shown to enhance the efficacy of various oncolytic vectors. Expression of the p14 FAST protein from an oncolytic vesicular stomatitis virus (VSVΔ51FAST) enhanced the therapeutic efficacy of the virus, and co-delivery of VSVΔ51FAST with an oncolytic vaccinia virus also provided a synergistic effect in cell culture and ex vivo models of cancer [19,20]. VSV-encoding FAST proteins also showed enhanced efficacy in combination with natural killer T (NKT) cell activation therapy in tissue culture and mouse models of metastatic breast cancer [21]. A replication-competent retrovirus vector also showed greater cytotoxicity in cancer cells in culture when FAST proteins were encoded within the vector [22]. Expression of the p14 FAST protein from an early region 1 (E1)-deleted, replication-defective HAdV (AdFAST) in human and mouse in vitro cancer models led to extensive cell fusion, loss of cellular metabolic activity, and induction of apoptosis-like cell death [23,24]. Unfortunately, the very promising results for AdFAST in vitro did not translate in vivo, where no effect on tumor growth or mouse survival was observed in either an immune-deficient human A549 lung adenocarcinoma xenograft or immune-competent 4T1 tumor model [23,24]. Expression of p14 FAST protein from a CRAd led to enhanced vector efficacy due to increased expression of the protein during viral replication within infected cells [25]. In human A549 lung adenocarcinoma cells in culture, CRAdFAST induced monolayer fusion, reduced cellular metabolic activity, and induced robust cleavage of caspase 3, suggesting the cells had undergone an apoptosis-like cell death. In an A549 xenograft model of cancer in immunodeficient mice, CRAdFAST induced substantial syncytium formation, which led to large acellular regions within treated tumors and significantly reduced the tumor growth rate compared to CRAd vector-lacking p14 FAST. Despite these positive indicators, CRAdFAST did not lead to complete tumor regression, suggesting that alternative approaches may be needed to augment the anti-cancer effects of the p14 FAST protein.

Most HAdV-based vectors, and many CRAd, lack the viral early region 3 (E3). E3 is not required for virus replication in culture, and removal of E3 increases the cloning capacity of the vector to about 8 kb of foreign DNA for E1/E3-deleted vectors [26,27]. Several studies have shown that inclusion of all or part of the E3 region can enhance CRAd efficacy [28,29,30,31]. The E3 region encodes a number of immunoregulatory proteins, which may allow CRAd to persist and replicate for longer periods within the tumor before being eliminated by the host immune system [30]. The E3 region also encodes the adenovirus death protein (ADP), an 11.6 kDa glycoprotein that localizes to the nuclear membrane in infected cells and is required for efficient cell lysis and release of progeny virions [32]. Although ADP is encoded within the E3 region, it is minimally expressed during early times of infection from the E3 promoter but is produced at a high level from the major late promoter (MLP) late in infection [33]. ADP-deleted viruses have a small plaque morphology, consistent with impaired cell lysis, progeny release, and spread of virus to adjacent cells [32,34]. Oncolytic HAdV that overexpress ADP alone, in the absence of other E3 proteins, show enhanced efficacy in vitro and in vivo [35,36,37,38,39,40,41]. Since fusogenic HAdV do not spread between nuclei of a syncytium [42], we hypothesize that p14 FAST protein-mediated cellular fusion followed by ADP-mediated nuclear lysis may elicit a synergetic anti-cancer effect to enhance virus spread through the tumor. In this study, we have examined the ability of p14 FAST protein and ADP to act synergistically to enhance CRAd efficacy in a tissue culture model of cancer.

## 2. Results

### 2.1. Construction and Characterization of Oncolytic Vectors Expressing p14 FAST Protein and E3

**Figure 1:**
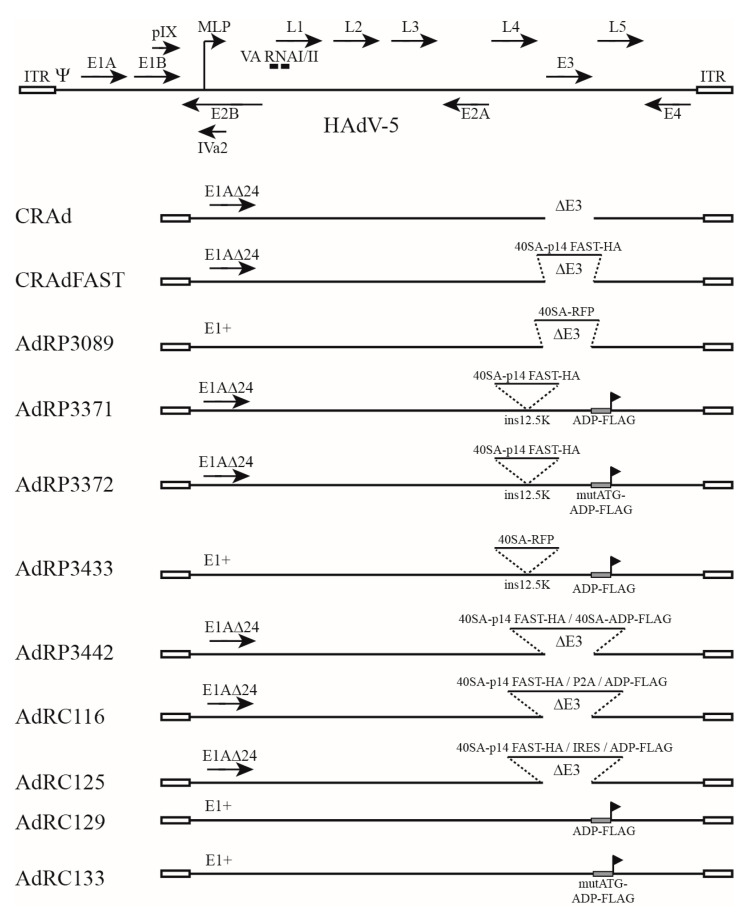
Viral constructs used in this study. A simplified transcription map for HAdV-5 is also shown, with the black arrows representing the indicated transcription unit. E—early transcription unit. L—late transcription unit. ITR—inverted terminal repeat. MLP—major late promoter. E1AΔ24—24-bp deletion in the conserved region 2 (CR2) of the E1A gene. 40SA—splice acceptor site derived from the HAdV-40 long fiber transcript. FAST—fusion-associated small transmembrane. HA—hemagglutinin epitope tag. RFP—red fluorescent protein. ins12.5K—transgene insertion within the E3 12.5K open reading frame. ADP—adenovirus death protein. FLAG—FLAG epitope tag. mutATG—mutation of the ADP start codon from ATG to TAG. P2A—self-cleaving peptide derived from porcine teschovirus-1. IRES—internal ribosome entry site derived from encephalomyocarditis virus (EMCV). Please note that the maps are not drawn to scale.

Our initial oncolytic vector armed with p14 FAST, CRAdFAST, showed an excellent ability to fuse cells and enhance cell killing in cancer cells in culture [25,43], but mediated only a modest improvement in anti-cancer effect on tumor growth in vivo [25]. Given that several studies have shown that inclusion of all or part of the E3 region can enhance CRAd efficacy [28,29,30,31,35,36,37,38,39,40,41], we investigated several methods to permit efficient co-expression of the p14 FAST protein and E3. Indeed, in a previous study, we showed that co-infection of CRAdFAST and an HAdV-based vector-encoding E3 showed an enhanced ability to kill A549 cells compared to co-infection of CRAdFAST and an HAdV-based vector-lacking E3 [43]. CRAdFAST encodes the p14 FAST gene with an upstream splice acceptor site derived from the HAdV-40 long fiber transcript (herein termed 40SA) inserted in place of the E3 region, to allow for replication-dependent expression of the p14 FAST protein from the viral MLP [25]. Specifically, the vector is deleted of two BglII fragments spanning 28,134 and 30,819 bp of the HAdV-5 genome, thus placing the p14 FAST expression cassette ~235 bp downstream of the E3 12.5K start codon. E3 12.5K is a relatively uncharacterized protein of unknown function [44,45]. To retain high-level p14 FAST protein expression and permit expression of most E3 genes under native regulation, we simply re-introduced the missing BglII-E3 fragments downstream of p14 FAST in our oncolytic vector, generating AdRP3371 (Figure 1). Thus, this vector has the entire E3 region reintroduced in its native position, but E3 12.5K is insertionally inactivated due to the presence of the 40SA-p14 FAST cassette. Since all of the relevant E3 splicing signals are retained in this construct, it was hoped that the remaining six proteins encoded within the E3 region (E3-6.7K, E3-gp19K, ADP, RIDα, RIDβ, and E3-14.7K) might be expressed temporally at appropriate levels. We included a C-terminal FLAG tag on the ADP to more easily monitor expression of this protein as a surrogate marker for E3. AdRP3372 is similar in structure to AdRP3371 but is mutated in the start codon of ADP and thus does not express the protein.

**Figure 2:**
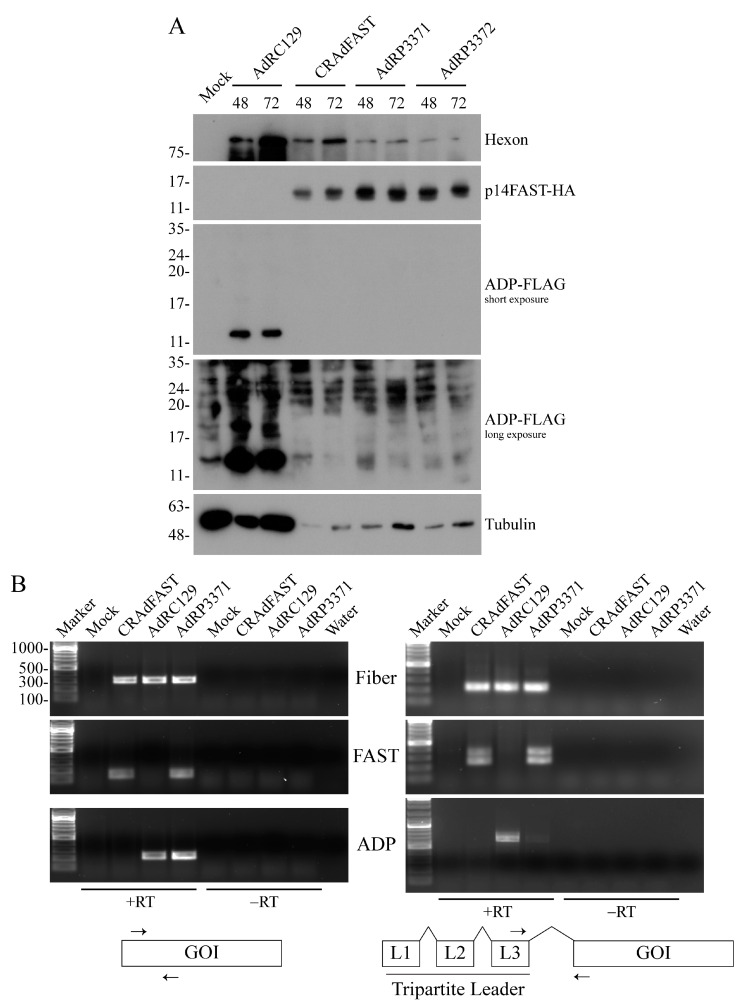
Insertion of a splice acceptor-driven p14 FAST expression cassette in the intact E3 region inhibits efficient splicing of downstream E3 transcripts. Panel (**A**): A549 cells were infected at an MOI of 1 with AdRC129, CRAdFAST, AdRP3371, or AdRP3372, and crude cellular lysates were collected at 48 and 72 h post infection (hpi). The resulting proteins were analyzed by immunoblot for HAdV-5 hexon, p14 FAST protein (HA-tagged), ADP (FLAG-tagged), and tubulin. Panel (**B**): A549 cells were infected with CRAdFAST, AdRC129, or AdRP3371 at an MOI of 3 PFU/cell; total RNA was isolated 48 h later and was converted to cDNA. Samples were analyzed by conventional PCR for the total (**left panel**) fiber, p14 FAST or ADP, or using oligonucleotide primers designed to amplify these genes spliced to the HAdV-5 tripartite leader (**right panel**) and visualized by agarose gel electrophoresis. A schematic of the primer-binding sites (indicated with black arrows) is also shown. GOI = gene of interest (fiber, p14 FAST, or ADP).

We first examined the expression of viral and transgene proteins from our vectors. A549 cells were infected at an MOI of 1 with AdRC129, CRAdFAST, AdRP3371, and AdRP3372, and isolated total cellular proteins 48 and 72 h post infection (hpi) for immunoblot analysis. The cellular lysates were prepared by carefully discarding the medium and overlaying the monolayer with protein-loading buffer. As shown in Figure 2A, the levels of tubulin were significantly reduced for all of the vectors that expressed the p14 FAST protein, which is addressed in detail below. All of the p14 FAST protein-expressing viruses produced similar quantities of the p14 FAST protein. Expression of ADP was clearly evident from AdRC129, appearing as a ~14 kDa band, with an additional ~18 kDa species evident on long exposure. AdRP3371 did not appear to produce the FLAG-tagged ADP. On long exposure, signal was detected in the region of the autoradiograph where ADP would be expected, but a similar signal was observed for AdRP3372, which cannot express the protein due to mutation of the ADP start codon. Thus, AdRP3371 does not produce detectable levels of ADP.

To gain insight into the potential cause for the apparent lack of expression of ADP from AdRP3371, we performed reverse transcriptase-PCR on RNA isolated from AdRP3371- or control virus-infected A549 cells. Briefly, A549 cells were infected with CRAdFAST, AdRC129, or AdRP3371 at an MOI of 3 and, at 24 hpi, total RNA was isolated, converted to cDNA, and analyzed for total fiber, p14 FAST, or ADP transcript, or for a spliced product comprising the L3 region of the tripartite leader and fiber, p14 FAST, or ADP, similar to previously described [43]. As shown in Figure 2B, analysis of fiber transcripts showed a similar level of total and spliced transcript for all three viruses, with no signal present in the absence of reverse transcription. For p14 FAST, we again observed similar levels of total and spliced transcript for both CRAdFAST and AdRP3371, consistent with the similar level of p14 FAST protein produced from the two viruses. For the L3-p14 FAST PCR reaction, we observed two products. Sequencing of the two PCR fragments showed that the lower band was identical in sequence to that predicted for accurate splicing of the tripartite leader directly onto p14 FAST, whereas the larger-sized L3-p14 FAST contained an additional 106 bp derived from 27,834 to 27,939 of the HAdV-5 genome, similar to as we have previously observed [43]. This segment corresponds in part to the x leader fragment that is variably attached to some mature transcripts originating from the MLP [46,47,48,49]. For ADP, although a similar level of total ADP transcript was observed for AdRC129- and AdRP3371-infected cells, AdRP3371 appeared to yield a significantly reduced quantity of the L3-ADP spliced transcript. Sequence analysis of the L3-ADP PCR products from AdRC129- and AdRP3371-infected cells showed that they are identical to spliced transcripts normally produced during HAdV infection [47] and that would be expected to give rise to ADP protein. We did not detect aberrant splice products in this analysis. Thus, based on the presence of appropriate splice products, AdRP3371 would be expected to produce some ADP protein, but fails to produce the protein at detectable levels. We did not examine whether other E3 proteins were produced from this virus but assume that some or all E3 proteins may be similarly adversely affected by the upstream p14 FAST expression cassette. Thus, although this particular vector configuration allows for significant expression of p14 FAST protein, it does not permit expression of all E3 proteins, at least not at a detectable level.

**Figure 3:**
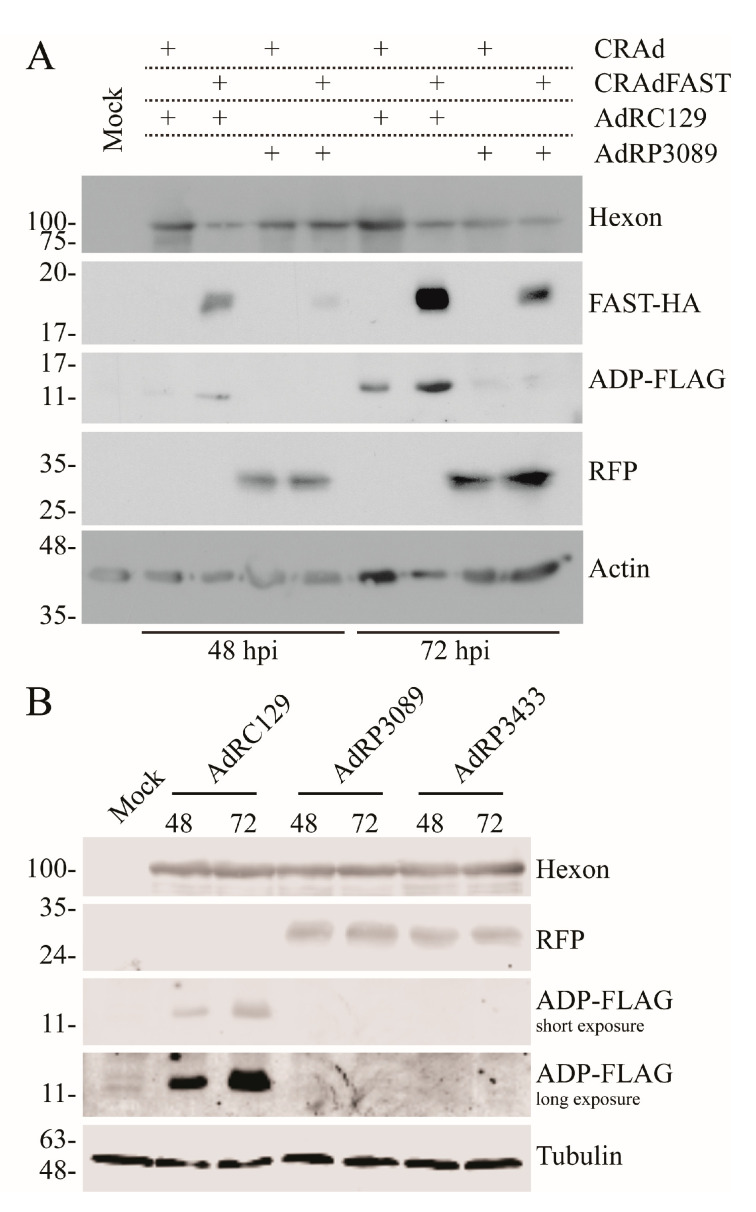
Insertion of an exogenous splice acceptor-driven expression cassette in the intact E3 region inhibits efficient expression of E3 proteins. Panel (**A**): A549 cells were infected at an MOI of 1 with CRAd or CRAdFAST in combination with either AdRC129 or AdRP3089, and crude cellular lysates were collected at 48 and 72 hpi. The resulting proteins were analyzed by immunoblot for HAdV-5 hexon, p14 FAST protein (HA-tagged), ADP (FLAG-tagged), RFP, and actin. Panel (**B**): A549 cells were infected at an MOI of 1 with AdRC129, AdRP3089, and AdRP3433, and crude cellular lysates were collected at 48 and 72 hpi. The resulting proteins were analyzed by immunoblot for HAdV-5 hexon, RFP, ADP (FLAG-tagged), and tubulin.

Lack of expression of ADP could be due to either deleterious effects within the cell caused by expression of p14 FAST protein, or to limitations imposed by the specific configuration of the expression cassettes within the vector. To address the first possibility, we co-infected cells with CRAd either expressing or lacking the p14 FAST gene along with a second vector-expressing ADP (AdRC129) or RFP (AdRP3089), both at an MOI of 1. AdRP3089 expresses the monomeric RFP protein from a 40SA expression cassette replacing the E3 region, and thus expresses RFP “late” within the virus lifecycle [50]. Protein expression was examined in crude protein samples collected at 48 and 72 hpi. As shown in Figure 3A, when expressed from different vectors, the quantity of either ADP or RFP appeared unaffected by the co-expression of the p14 FAST protein. Thus, although the p14 FAST protein can ultimately compromise cellular metabolic function [23,24,25], it does not appear to adversely impact expression of other virus-encoded proteins, at least within the time frame examined. This data suggests that the absence of expression of ADP from AdRP3371 may be due to the specific configuration of the expression cassettes within the vector.

We next examined whether lack of expression of the downstream E3 proteins was due to the presence of specifically the p14 FAST gene, which could be acting to promote cryptic or non-canonical splicing [51], ultimately preventing expression of the downstream E3 genes. We created a virus similar to AdRP3371 but containing the 40SA-RFP gene replacing the 40SA-p14 FAST gene, which was designated AdRP3433. Analysis of protein expression from AdRP3433 showed that RFP was efficiently expressed and at a similar level as our previously characterized virus, AdRP3089 [50], which has the entire E3 region replaced by an 40SA-RFP expression cassette (Figure 3B). Similar to AdRP3371, we did not observe expression of the downstream FLAG-tagged ADP gene from AdRP3433, suggesting that insertion of any 40SA gene at this position (~28,134 bp of the HAdV-5 genome) adversely affects expression of E3.

### 2.2. Construction and Characterization of Oncolytic Vectors Expressing p14 FAST Protein and ADP Through Tandem Splice Acceptor Cassettes

**Figure 4:**
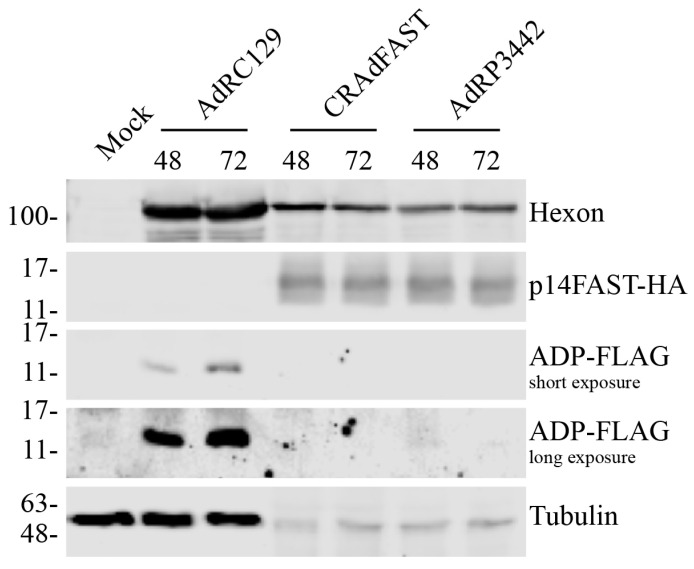
Inclusion of a tandem 40SA-p14 FAST/40SA-ADP cassette replacing the E3 region does not permit expression of ADP. A549 cells infected at an MOI of 1 with AdRC129, CRAdFAST, or AdRP3442 were harvested at 48 and 72 hpi, and protein samples were analyzed by immunoblot for HAdV-5 hexon, p14 FAST protein (HA-tagged), ADP (FLAG-tagged), and tubulin.

Several studies showed that the efficacy of an oncolytic vector can be enhanced through expression of the E3-encoded ADP, in the absence of other E3 proteins [35,36,37,38,39,40,41]. Although the specific mechanism of action is unclear, ADP aids in mediating cell death and release of virus from the infected cells [52]. Since placement of the 40SA-p14 FAST cassette within the region normally occupied by E3 allowed for high-level, replication-dependent expression of the p14 FAST protein [25], we asked if simply inserting a second 40SA-ADP cassette downstream of the p14 FAST gene would allow for high-level expression of both proteins. Of note, our 40SA contains a branchpoint consensus sequence and a polypyrimidine tract upstream of the SA [25,53], which are required for efficient splicing. To test protein expression from this vector, A549 cells were infected with AdRC129, CRAdFAST, and AdRP3442 (a CRAd with the tandem 40SA-p14 FAST/40SA-ADP cassette replacing the E3 region) at an MOI of 1, and crude protein lysates were collected 48 and 72 h later. As shown in Figure 4, once again we observed low levels of tubulin and hexon for the viruses expressing the p14 FAST protein. Both CRAdFAST and AdRP3442 produced similar quantities of p14 FAST protein. Although a weak signal was observed for AdRP3442 at approximately the size of ADP on long exposure, it is likely due to non-specific antibody binding as a similar signal was observed in mock infected cells. Thus, although AdRP3442 is capable of producing significant levels of p14 FAST protein from a splice acceptor cassette contained in the E3 region, no expression was detected from a second splice acceptor cassette located downstream of the p14 FAST gene.

**Figure 5:**
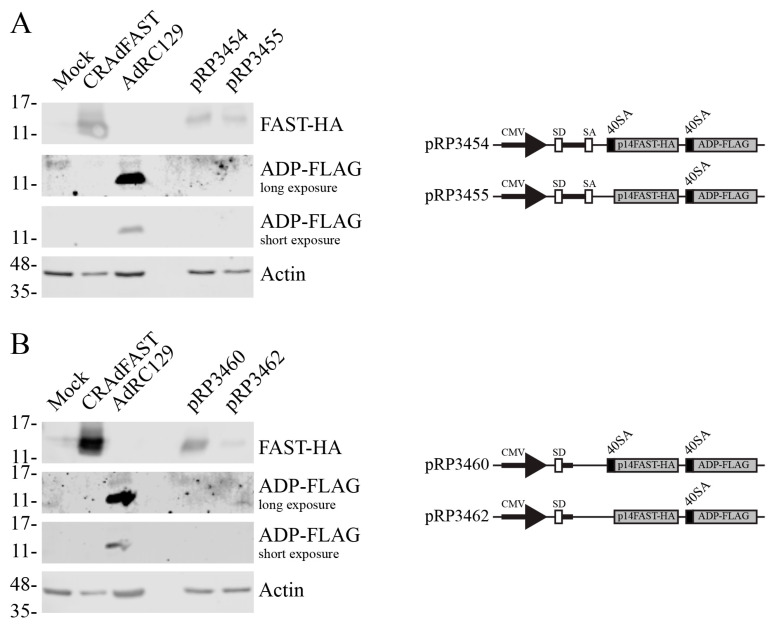
Removal of the upstream splice acceptor from the tandem 40SA-p14 FAST/40SA-ADP cassette does not lead to expression of ADP. Panel (**A**): A549 cells were transfected with pCI-neo-based plasmid containing 40SA-p14 FAST/40SA-ADP (pRP3454) or p14 FAST/40SA-ADP (pRP3455), and expression of p14 FAST (HA-tagged) and ADP (FLAG-tagged) was assessed 24 h later. Panel (**B**): A549 cells were transfected with pCI-neo-based plasmid deleted of the splice acceptor site located in the optimized chimeric intron normally contained in this plasmid, and containing 40SA-p14 FAST/40SA-ADP (pRP3460) or p14 FAST/40SA-ADP (pRP3462), and expression of p14 FAST (HA-tagged) and ADP (FLAG-tagged) was assessed 24 h later. Schematic representations of the various plasmids are also shown.

We next addressed whether the upstream splice acceptor site in the 40SA-tandem cassette was somehow occluding the downstream splice acceptor site. We constructed plasmids based on pCI-neo that contained the 40SA-ADP gene downstream of a p14 FAST gene that contained or lacked the 40SA site. pCI-neo naturally contains an optimized chimeric intron (donor, branchpoint, and acceptor sites) downstream of the CMV enhancer/promoter region [54]. We would predict that the chimeric splice donor may variably combine with one of the three available downstream splice acceptors in the plasmid, giving rise to both the p14 FAST protein and ADP. These plasmids were transfected into A549 cells, and 24 h later the resulting crude cellular lysates were analyzed for the quantity of protein by immunoblot. As positive controls, we also included extracts from cells infected with virus expressing the HA-tagged p14 FAST protein or FLAG-tagged ADP on the gel. As shown in Figure 5A, plasmid that did (pRP3454) or did not (pRP3455) encode the 40SA directly upstream of the p14 FAST gene expressed high levels of the protein. This is unsurprising since the native SA from the chimeric intron is present in this construct, which should allow for efficient expression of the p14 FAST gene. However, ADP expression was not observed from either of these constructs. We made additional plasmid constructs that lacked the native SA in the chimeric intron of pCI-neo, such that the only SA were immediately upstream of the p14 FAST or ADP genes. Once again, although we observed significant expression of p14 FAST protein either in the presence (pRP3460) or absence (pRP3462) of its immediate upstream SA, we did not observe the expression of ADP from either construct (Figure 5B). Thus, it appears that the 40SA immediately upstream of ADP in these constructs is not in an appropriate context to be efficiently recognized by the cellular splicing machinery. Therefore, use of two tandem genes containing 40SA to achieve coexpression of proteins from the E3 region of HAdV vectors is not a viable approach.

### 2.3. Construction and Characterization of Oncolytic Vectors Expressing p14 FAST Protein and ADP Through Bicistronic Cassettes

Since the use of two tandem expression cassettes for p14 FAST protein and ADP expression did not yield significant expression of the downstream transgene, we explored two common methods to achieve bicistronic expression of the p14 FAST protein and ADP. First, we developed a bicistronic cassette in which the two genes were separated by a 2A ribosomal skipping motif derived from porcine teschovirus-1 (P2A) [55,56]. The p14 FAST-P2A-ADP expression cassette was placed behind the 40SA splice acceptor site and was used to replace the E3-region in a CRAd vector, generating AdRC116 (Figure 1). Second, we developed a bicistronic cassette in which the two genes were separated by an internal ribosome entry site (IRES) derived from encephalomyocarditis virus (EMCV) [57] and, again, the p14 FAST/IRES/ADP expression cassette was placed behind the splice acceptor site and was used to replace the E3-region in a CRAd vector, generating AdRC125.

**Figure 6:**
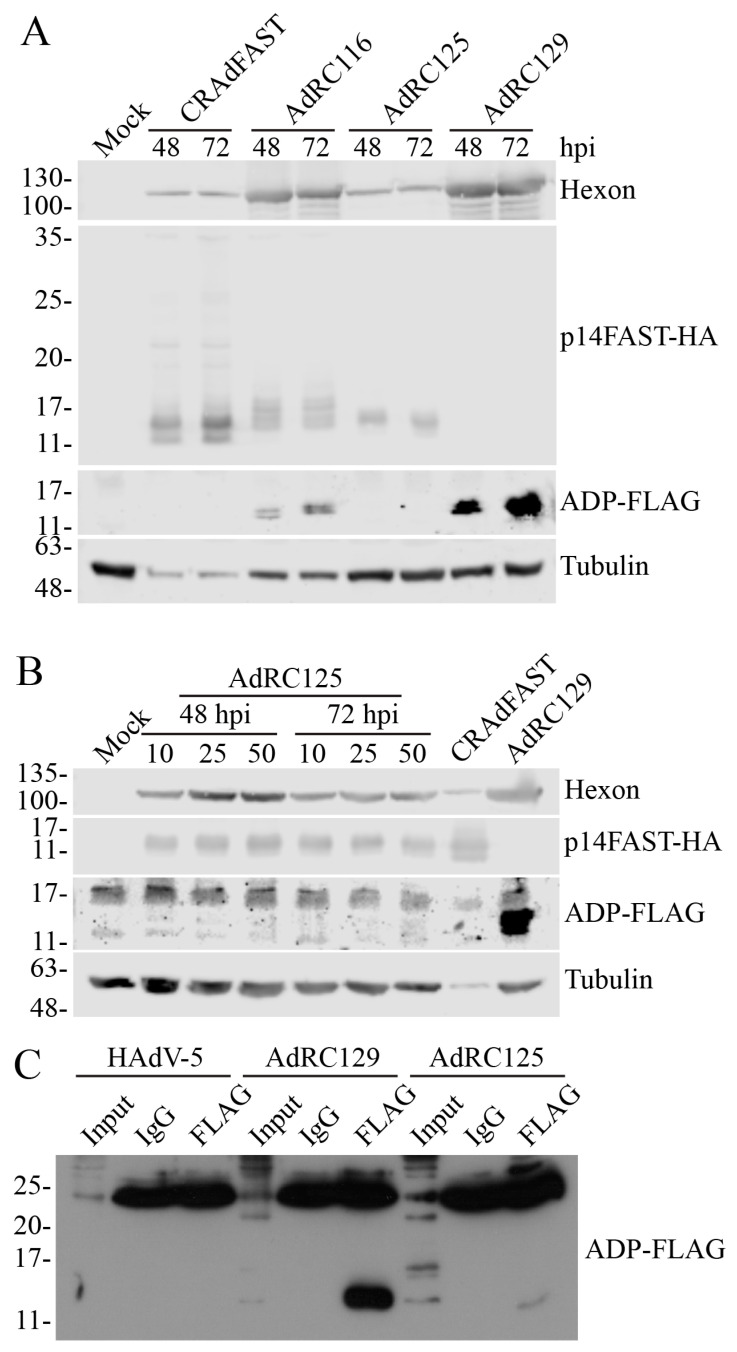
Expression of p14 FAST protein and ADP from bicistronic expression constructs encoded within CRAd. Panel (**A**): A549 cells were infected at an MOI of 1 with CRAdFAST, AdRC116, AdRC125, or AdRC129. Crude cellular lysates were harvested at 48 and 72 hpi and analyzed by immunoblot for HAdV-5 hexon, p14 FAST protein (HA-tagged), ADP (FLAG-tagged), and tubulin. Panel (**B**): A549 cells were infected at varying MOI (10, 25, or 50) with AdRC125, and crude protein extracts were isolated at 48 and 72 hpi. In parallel, A549 cells were infected (MOI of 1) with CRAdFAST or AdRC129, and crude cell extracts were isolated at 72 hpi. The protein extracts were analyzed by immunoblot for HAdV-5 hexon, p14 FAST protein (HA-tagged), ADP (FLAG-tagged), and tubulin. Panel (**C**): A549 cells were infected with HAdV-5, AdRC129, or AdRC125 at an MOI of 10. Crude protein extracts were prepared in modified RIPA buffer and subjected to immunoprecipitation with antibody to FLAG or IgG. The resulting proteins and sample of input were subjected to immunoblot for FLAG.

To assess whether these new vectors efficiently co-expressed p14 FAST protein and ADP, A549 cells were infected at an MOI of 1 with CRAdFAST, AdRC116, AdRC125, or AdRC129, and protein was collected for immunoblot at 48 and 72 hpi. As shown in Figure 6A, both AdRC116 and AdRC125 allowed for expression of p14 FAST protein, although the quantity of protein produced from these vectors appeared lower than for CRAdFAST. Indeed, when normalized to hexon protein expression, p14 FAST protein appears to be produced ~10-fold less efficiently from the bicistronic vectors relative to CRAdFAST. The HA epitope tag that we fused to p14 FAST for ease of immunodetection is known to be cleaved during apoptotic cell death by caspases 3 and 7 [58]. HAdV [59], ADP [38], and p14 FAST protein [25,60] are all known to induce apoptotic-like cell death, suggesting that reduced detection of p14 FAST protein from AdRC116 could be due to cleavage of the tag from p14 FAST, rather than true decreased expression. However, co-infection of vector-expressing p14 FAST-HA (CRAdFAST) with virus-expressing ADP (AdRC129) did not lead to apparent reduced expression of p14-FAST-HA protein relative to cells infected with CRAdFAST and virus lacking ADP (AdRP3089) (Figure 3A). Thus, the reduced levels of p14 FAST-HA observed in AdRC116- and AdRC125-infected cells is likely due to true reduced expression of these genes. p14 FAST protein expressed from AdRC116 migrated as a slightly larger species than that produced from CRAdFAST. Since P2A is “cleaved” after the c-terminal glycine of the recognition motif [55], 21 amino acids derived from the motif are retained on the C-terminus of the p14 FAST protein, resulting in the observed shift in protein migration. The presence of this extra small peptide on the carboxy terminus of the p14 FAST protein is not expected to affect protein function, as we showed that the addition of an HA tag to this region does not have any discernable effect on the ability of the protein to mediate fusion [24]. Although ADP has a predicted size of 10.5 kDa in HAdV-5 [61], the protein is post-translationally modified (i.e., glycosylation and palmitoylation, [52]) resulting in proteins that migrate as ~14–30 kDa on SDS-PAGE [62]. For AdRC129 (an otherwise wildtype HAdV-5 with a FLAG-epitope tag on ADP), we detected a protein doublet of ~14 kDa. We observed similar-sized bands for AdRC116, although the intensity was approximately one-tenth that observed for AdRC129 at both time points examined. No FLAG signal was noted for AdRC125 at an MOI of 1. Infection of A549 cells with AdRC125 at higher MOI did yield a very weak signal at ~14 kDa at the highest MOI examined at the 72 hpi time point (Figure 6B). The weak expression of ADP from AdRC125 was also detected by immunoprecipitation/immunoblot (Figure 6C). These results confirm that the two CRAdFAST/ADP vectors can replicate in A549 cells and that the bicistronic expression cassette provide co-expression of p14 FAST protein and ADP. However, with the expression cassette configurations in our vectors, p14 FAST protein expression was reduced compared to our original p14 FAST protein-expressing vector CRAdFAST, and we were not able to achieve a level of ADP expression similar to the wildtype virus. Given the very poor expression of ADP from AdRC125, we did not characterize this vector further.

### 2.4. p14 FAST Protein Impact on Cytoskeletal and Histone H3 Proteins

**Figure 7:**
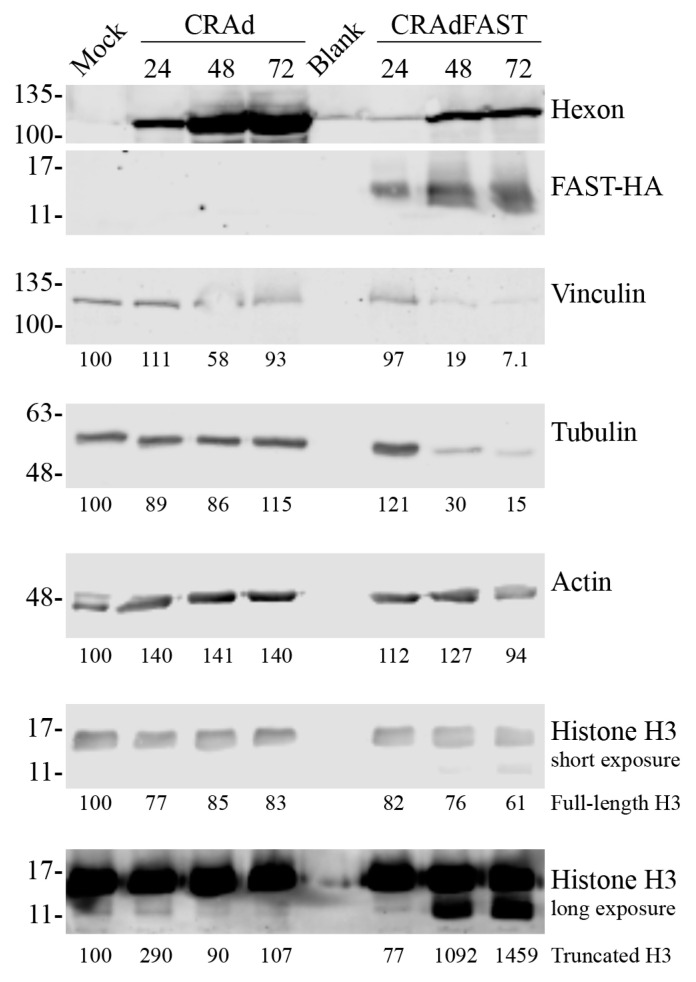
Immunoblot analysis of common loading control proteins in A549 cells infected with CRAdFAST. A549 cells were infected (MOI of 1) with CRAd or CRAdFAST. At varying times post-infection (24, 48, and 72 h), the cells were collected into the medium, cells and debris were pelleted by centrifugation at 10,000× *g*, and the resulting pellet was resuspended in 2× protein-loading buffer. The proteins were subsequently analyzed by immunoblot using antibodies to HAdV-5 hexon, p14 FAST (HA-tagged), vinculin, tubulin, actin, and histone H3. The immunoblots were processed using an Odyssey CLx imaging system (LI-COR), and the resulting quantified band intensities are provided below each panel of the figure.

Many research groups probe for cytoskeletal proteins by immunoblot to control for differences in protein loading. In Figure 2A, we included tubulin as a loading control, and clearly showed that the level of tubulin protein within the cells infected with p14 FAST-expressing HAdV vectors was greatly reduced relative to cells infected with vector-lacking p14 FAST. Fusion of cells can result in formation of semi-adherent cell spheres that can release into the medium [24,25] and may be lost during cell/protein isolation, which would correspondingly reduce the signal of the loading control. However, cells treated with CRAdFAST did not appear to show as dramatic a reduction in actin signal (Figure 3A). To examine this phenomenon more directly, we infected A549 cells with CRAd or CRAdFAST, isolated protein at 24, 48, and 72 hpi, and determined the quantity of several proteins commonly used as loading controls. Infection of A549 cells with CRAd led to only minor changes in the quantity of most markers examined (Figure 7). In contrast, infection of A549 cells with CRAdFAST led to a ~10-fold reduction in vinculin and tubulin within the cells beginning at 48 hpi. Both actin and histone H3 showed a more modest decline in level in the CRAdFAST-treated cells, although a prominent, truncated form of histone H3 was observed in the CRAdFAST-treated cells. Thus, expression of the p14 FAST protein dramatically alters the level of some cytoskeletal proteins within the cell, which may promote the fusion process or contribute to the semi-adherent cell spheres frequently observed in fused monolayers.

### 2.5. AdRC116 Plaque Morphology in A549 Cells in Culture

**Figure 8:**
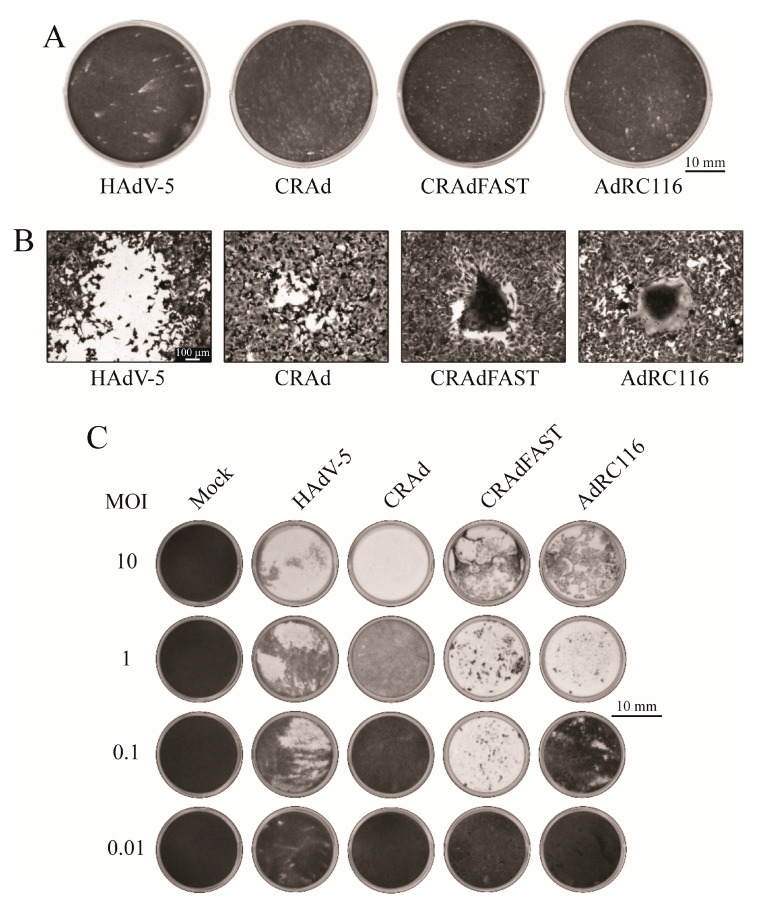
Analysis of viral plaque morphology and spread in tissue culture. Panel (**A**): A549 cells were infected with 10-fold serial dilutions of HAdV-5, CRAd, CRAdFAST, or AdRC116, and overlayed with medium supplemented with carboxymethylcellulose (CMC). Seven days later, the CMC medium was removed, and the monolayer was stained with crystal violet. Panel (**B**): High magnification bright-field microscopy images of plaques from Panel (**A**). Panel (**C**): A549 cells were infected with HAdV-5, CRAd, CRAdFAST, or AdRC116 at an MOI ranging from 0.01 to 10. Infected cells were fixed and stained with crystal violet 7 dpi. Three independent experiments were performed, and representative results are shown.

ADP significantly affects plaque size and morphology [34]. Thus, we would predict that AdRC116 would also exhibit altered plaque morphology relative to CRAdFAST. Plaque size and morphology was analyzed in A549 cells for the various viruses using a semi-viscous carboxymethylcellulose overlay to examine the efficiency of viral spread to adjacent cells [63]. Consistent with previous work [32,34,36,43], by 7 dpi the E3-expressing wildtype HAdV-5 formed large, comet-shaped plaques whereas the E3-deleted, non-fusogenic CRAd showed a small plaque morphology (Figure 8A). CRAdFAST also exhibited a small plaque phenotype, but these plaques showed clear evidence of cell fusion (Figure 8B). For AdRC116, ADP expression did not appear to significantly alter the plaque size or morphology relative to CRAdFAST, suggesting that p14 FAST protein expression provides the “dominant” phenotype to this virus. Thus, ADP increased plaque size of non-fusogenic HAdV but did not affect plaque morphology of fusogenic CRAd, at least based on the level of ADP expression we achieved with AdRC116.

We also examined the impact of ADP expression from AdRP116 on virus spread through a cytopathic effect (CPE) assay. When delivered at low MOI, the virus must undergo multiple rounds of infection, replication, lysis, and spread to achieve a complete CPE of a monolayer of cells. Virus with a good ability to spread would require a lower MOI to achieve a complete monolayer CPE, whereas virus with a poor ability to spread would require a higher MOI to achieve a complete CPE. A549 cells were infected at a MOI ranging from 0.01–10 plaque-forming units (PFU)/cell with HAdV-5, CRAd, CRAdFAST, or AdRC116, overlayed with liquid medium, and stained with crystal violet at 7 dpi. HAdV-5 caused a significant CPE at an MOI of 1, and exhibited greater potency than the ADP-deleted, non-fusogenic CRAd (Figure 8C). CRAdFAST mediated a complete CPE at an MOI of 0.1, suggesting greater inherent cytotoxicity or ability to spread relative to HAdV-5 and CRAd. AdRC116 achieved a complete CPE at an MOI of 1, suggesting slightly reduced cytotoxicity compared to CRAdFAST. At an MOI of 0.1, the monolayer for AdRC116-infected cells appeared extensively fused, as assessed by phase-contrast microscopy, but remained relatively intact. The reduced cytotoxicity of AdRC116 is likely due to the reduced expression of p14 FAST protein from this virus relative to CRAdFAST (Figure 6). This latter observation also suggests that the quantity of p14 FAST-HA protein detected by immunoblot is reflective of the fusion ability of the virus, and that caspase-mediated cleavage of the HA tag from the p14 FAST is likely not occurring at an appreciable level in our hands. Thus, as in our plaque assay, co-expression of ADP did not increase spread of CRAd-expressing p14 FAST protein and, indeed, the FAST protein appears to provide the dominant phenotype for both plaque size and virus spread.

### 2.6. Analysis of AdRC116-Mediated Cell Killing in A549 Cells in Culture

**Figure 9:**
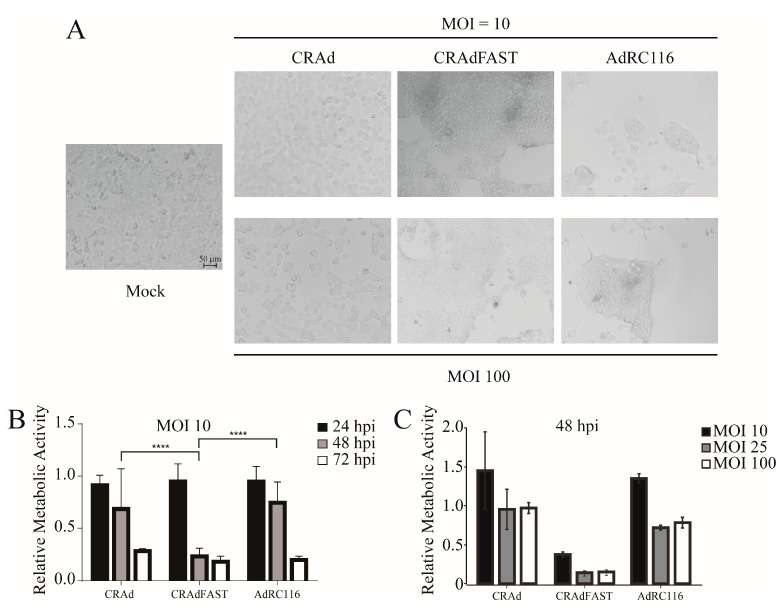
Analysis of AdRC116-mediated cell killing in A549 cells in culture. Panel (**A**): A549 cells were infected at an MOI of 10 or 100 with CRAd, CRAdFAST, or AdRC116 (or mock-infected) and images were obtained 48 h later. Panel (**B**): A549 cells were infected at an MOI of 10 with CRAd, CRAdFAST, or AdRC116 (or mock-infected) and subjected to an MTS assay at 24-h intervals. Values were normalized to mock-infected cells. Panel (**C**): A549 cells were infected with varying MOI of CRAd, CRAdFAST, or AdRC116 and subjected to MTS assay at 48 hpi. Values were normalized to mock-infected cells. For Panels (**B**,**C**), the graphed values are the average with error bars representing standard deviation, and three independent experiments were performed in triplicate. **** *p* < 0.0001.

We previously demonstrated that expression of the p14 FAST protein in A549 cells from either a replication-defective [24] or replication-competent [25] HAdV-based vector resulted in a significant reduction in cellular metabolic activity and, ultimately, activation of caspase 3 and apoptotic cell death. Therefore, we next tested whether co-expression of p14 FAST protein and ADP from AdRC116 elicited enhanced effects on cellular metabolic activity relative to CRAdFAST, assessing whether AdRC116 adversely impacted cell health either more rapidly or at a lower MOI. Cells were infected with our various viruses at an MOI of 10 or 100, and cell morphology was assessed 48 h later. Infection of cells with CRAd resulted in a CPE that was much more pronounced at an MOI of 100 (Figure 9A). Infection of cells with CRAdFAST caused extensive cell fusion and a CPE at both MOI examined. Infection with AdRC116 also caused extensive syncytium formation and a CPE, but the fused cells were in smaller patches relative to CRAdFAST-treated cells. Thus, co-expression of p14 FAST and ADP does appear to substantially alter the nature of the CPE in A549 cells relative to expression of p14 FAST alone, once again, given the relative level of protein expression achieved in these constructs.

To determine whether AdRC116 reduced cell viability of A549 cells in culture, cells were treated with an MOI of 10 of the various viruses, and the cellular metabolic activity was examined at varying times post-infection. Treatment with CRAd or AdRC116 resulted in a decline in metabolic activity to approximately 80% and 30% of that observed for untreated cells at 48 and 72 hpi, respectively (Figure 9B). Treatment of A549 cells with CRAdFAST caused a significant reduction in metabolic activity relative to both CRAd or AdRC116 as early as the 48-hpi time point. We also examined the effect of MOI for each virus at a 48-hpi time point. Once again, CRAd and AdRC116 showed a similar effect at all MOI examined (Figure 9C). CRAdFAST again induced a more dramatic decline in cellular metabolic activity at all MOI. Taken together, these results suggest that the reduced level of expression of the p14 FAST protein from AdRC116 compromises the efficacy of the vector relative to CRAdFAST, and that co-expression of ADP cannot compensate for this, at least not at the level of ADP expression achieved from the AdRC116 vector.

## 3. Discussion

Although many HAdV-based vectors are deleted of E3 to increase the cloning capacity of the vector, previous work showed that inclusion of an intact E3 region can provide a benefit to oncolytic HAdV vectors [28,29,30,31]. For example, the E3 encoded ADP functions to enhance release of progeny virus at the very late stage of infection [32,45,64], which would be of obvious benefit to enhance the spread of oncolytic HAdV throughout a tumor. We thus asked whether co-expression of E3 or ADP alone could enhance the efficacy of cancer cell killing of an oncolytic vector expressing the p14 FAST fusogenic protein. While p14 FAST may aid spread of the virus (or the virus-mediated toxicity) among adjacent cells, E3 or ADP may aid in the release and broader dissemination of the virus within the tumor mass, conceivably providing a synergistic effect.

We first developed an oncolytic vector that retained the entire E3 region in its native position, but with the 40SA-p14 FAST expression cassette inserted within the coding sequence of the E3 12.5K protein, designated AdRP3371. The position of the p14 FAST expression cassette with respect to the E3 promoter and MLP is identical to that found in our original CRAdFAST [25], and indeed allowed for high-level expression of the p14 FAST protein from AdRP3371 (Figure 2). The 12.5K protein, which is dispensable for virus replication [44], is found in most mastadenoviruses [65,66], but its function remains unknown [44,45]. Although E3 12.5K is insertionally inactivated by the p14 FAST expression cassette, all native E3 cis-acting regulatory elements required for expression and splicing are retained in this construct, which should have allowed for expression of all other E3 ORFs. E3 transcription can occur early, originating from the E3 promoter, or late, originating from the MLP [67]. In both cases, the position of our p14 FAST ORF within E3 12.5K effectively places it within an intron for all other E3 ORF and should be spliced out from the other major E3 transcripts [47,68]. We did detect ADP transcripts originating from the MLP in AdRP3371-infected cells (Figure 2B), but not detectable ADP protein. The p14 FAST cassette does not contain a transcription termination signal, but there may be other cryptic regulatory elements present within the gene or flanking DNA that adversely affects transcription, splicing, or translation of the downstream E3 regions [51]. Inclusion of a 40SA-RFP expression cassette also inhibited expression of E3 proteins, indicating that the presence of the p14 FAST gene itself was not inherently responsible for inhibition of downstream splicing. The presence of the 40SA on the upstream p14 FAST cassette may act as a strong splicing signal that occludes or attenuates the use of downstream splice acceptors. However, in transient transfection assays of plasmid constructs, removal of the 40SA upstream of p14 FAST did not lead to usage of the downstream 40SA (Figure 5). The site of insertion within the 12.5K protein ORF may not be optimal, as there may be cis-acting elements present in the 12.5K protein ORF that influence splicing of the remainder of E3 and that are disrupted in our constructs. Splicing is a complex event that can be influenced by many subtle differences in RNA dynamics [69].

Several studies showed that the inclusion of ADP in an oncolytic vector can enhance cancer-killing efficacy in vitro and in vivo [35,36,37,38,39,40,41]. ADP is naturally expressed at low levels from the E3 promoter early in infection, but the levels of ADP are substantially increased late in infection as expression shifts to the MLP [62]. ADP is a type III membrane protein (i.e., N_endo_C_exo_) that undergoes extensive post-translational modification, including glycosylation [52]. As such, ADP is initially translated into the endoplasmic reticulum, where it undergoes N-glycosylation, then is transported to the trans-Golgi network where it undergoes extensive O-glycosylation [33]. ADP also appears to undergo proteolytic processing to remove a portion of the N-terminal region of the protein (including the glycosylated residues [33]), and it is also palmitoylated on the cytoplasmic tail [70]. Ultimately, the modified ADP protein localizes to the inner membrane of the nuclear envelope [33], where it promotes cell death, cell lysis, and release of the virus [32]. Cells infected with ADP-deficient virus remain viable for at least twice as long as those infected with ADP-encoding virus (5–6 days versus 2–3 days, respectively [34]), and ultimately produce small plaques [32,34].

Due to the extensive post-translational modification, ADP naturally migrates on SDS-PAGE as multiple species of approximate size 14K, 18–21K, and 27–31K [62]. Both AdRC129 and AdRC116 produced predominantly a single ADP species (or variably a doublet on some gels) of ~14 kDa (Figure 2 and Figure 6). Previous studies also showed that ADP can migrate as a single band when expressed from an otherwise E3-deficient oncolytic HAdV [38,71,72] and from wildtype virus [71]. We added a C-terminal FLAG tag to the protein expressed from our viruses, including AdRC129, which is an otherwise wildtype HAdV-5. Addition of the FLAG tag is not predicted to affect ADP function, as deletion of the C-terminal 22 amino acids of the HAdV-2 ADP does not alter the ability of the protein to mediate cell lysis [73]. The observation that AdRC129 is fully capable of producing comet-like plaques suggests that the FLAG tag does not affect the function of the protein.

We examined several methods to achieve expression of the p14 FAST protein and the E3-encoded ADP in our CRAd vectors. Since the 40SA-p14 FAST cassette provided very good, replication-dependent expression of p14 FAST from the E3 region (Figure 2 and ref. [25]), we explored whether inclusion of a second 40SA-expression cassette, placed downstream of p14 FAST, could mediate high-level expression of ADP. Unfortunately, although this vector produced high levels of the p14 FAST protein, it did not express detectable levels of ADP (Figure 4). Once again, perhaps the inclusion of an upstream 40SA, or other elements present in the construct, occludes the downstream splice acceptor. However, in similar plasmid-based constructs, removal of the upstream 40SA from p14 FAST did not lead to usage of the downstream 40SA on the ADP gene (Figure 5).

We explored two approaches to allow concomitant expression of the p14 FAST protein and ADP from a single cassette. The 2A self-cleaving peptides are derived from viral elements that allow for expression of multiple peptides from a single transcript through “skipping” of the peptide bond between a conserved proline and glycine residue in the 2A peptide [56]. We chose the 2A sequence derived from porcine teschovirus-1, as previous studies showed high efficiency of cleavage in human cells [74], and it has been used successfully in previous studies of oncolytic HAdV [55,75]. Both p14 FAST protein and ADP were expressed from AdRC116, although the quantity of these proteins produced from this vector was lower than our original CRAdFAST and wildtype HAdV, respectively (Figure 6A). It is not clear why p14 FAST protein was expressed at lower levels from AdRC116 compared to CRAdFAST. In both vectors, the p14 FAST gene was cloned into the identical position of the viral genome and expressed as part of the late transcription unit through inclusion of the 40SA. The HA epitope tag that we fused to p14 FAST can be cleaved during apoptotic cell death by caspases 3 and 7 [58], which could lead to an underestimation of the quantity of the p14 FAST protein produced from the vectors. The small amount of ADP produced from AdRC116 could enhance apoptosis within the infected cell [38,52], leading to greater removal of the HA tag. However, AdRC116 did not mediate enhanced cell death in our cytopathic effect assay (Figure 8B) or lead to enhanced cell death relative to CRAdFAST in our metabolic activity assays (Figure 9), indicating that this vector did not enhance apoptosis of cells and that the quantity of p14 FAST protein produced from AdRC116 is indeed low. Inefficient ribosomal skipping in the 2A peptide construct would lead to the production of a larger fusion protein [76], which would have led to a reduction in the full-length p14 FAST protein. However, we did not observe a large, uncleaved p14 FAST/ADP fusion protein product (Figure 6A), suggesting that the P2A cleavage event was quite efficient. Inherent properties of the bicistronic expression cassette used in AdRC116 may have led to the reduction in p14 FAST protein; perhaps the translation efficiency or stability of the bicistronic p14 FAST-P2A-ADP mRNA was reduced compared to the monocistronic p14 FAST protein.

We also examined co-expression of p14 FAST and ADP proteins using an IRES derived from EMCV, an approach which has also been used successfully in several previous oncolytic HAdV studies [75,77]. The mRNA of an IRES element is able to recruit ribosomes directly and allow for cap-independent translation [78]. Thus, in our construct, the upstream p14 FAST ORF and downstream ADP ORF would be translated in a cap-dependent and cap-independent manner, respectively. Once again, although the p14 FAST ORF in AdRC125 was in the same position as our original CRAdFAST vector, the quantity of p14 FAST protein produced from this vector was greatly reduced (Figure 6A,B). Moreover, we detected only limited expression of ADP from AdRC125 (Figure 6C), for reasons which are not clear. Previous studies showed that the use of the native start codon for the downstream gene in the EMCV IRES is crucial for optimal expression [79,80,81], and we indeed utilized this start codon for ADP. Several studies have suggested that the mRNA structure of the upstream gene can interfere with proper folding of the IRES RNA, thus adversely affecting the expression of the downstream gene, and that insertion of unstructured spacer sequences can improve the expression of the downstream gene [82,83]. However, since the upstream p14 FAST ORF also appears to be expressed poorly in this construct, we did not undertake additional effort to optimize this construct.

We hypothesized that syncytia induced with the p14 FAST protein combined with improved cell lysis mediated by ADP would lead to an enhanced ability to kill cancer cells. An examination of plaque morphology showed that fusion appeared to be the dominant phenotype for the vector expressing both the p14 FAST protein and ADP (Figure 8A,B). In a plaque assay, AdRC116 did not produce “comets” like wildtype HAdV-5, but instead produced small, fused regions of cells similar to CRAdFAST. We note that the level of ADP expression achieved from AdRC116 was ~10-fold lower than that typically observed for wildtype virus (Figure 6A), which likely contributed to the lack of comet-type release of virus. Alternatively, the process of cell fusion may significantly change the intracellular architecture and metabolism of the cell to the point where ADP can no longer efficiently perform its virus-release function.

Although both CRAdFAST and AdRC116 induced syncytia formation in A549 cancer cells (Figure 8A and Figure 9A), CRAdFAST was more efficient at reducing cell viability based on the MTS assay (Figure 9B,C). Previous work has shown that syncytium formation induced by FAST proteins ultimately triggers apoptosis and compromises membrane integrity in the dying cells [60]. Although AdRC116 was able to induce cell fusion (Figure 8B and Figure 9A), the reduced level of p14 FAST protein expressed from this virus (Figure 6A) may have led to the reduced effects on cell metabolic activity relative to CRAdFAST. We previously noted that the multiplicity of infection of both replication-competent and replication-defective HAdV vectors encoding p14 FAST protein, and thus absolute level of p14 FAST protein expression within the cell, can significantly affect cell fusion and metabolic activity, as well as cell membrane leakiness and induction of apoptosis [23,24,25]. Thus, the impact on cell viability within syncytia is directly proportional to the quantity of p14 FAST protein expressed within the fused cells.

One limitation to our work was that we evaluated expression from these vectors in a single cell line. A549 cells, a human lung adenocarcinoma cell line [84], is commonly used in studies of HAdV as it supports robust viral gene expression, replication, and virus yield. It is likely that different cell lines could yield varying results, perhaps resulting in greater expression of ADP or E3 proteins from vectors that failed to express in this study. Transcript splicing within the cell is a complex process that is influenced by many factors [85,86], and subtle differences between these proteins and processes between cell lines could influence splicing and expression from HAdV vectors.

In many of our immunoblot figures, we used tubulin as a “loading control” and noted that it was consistently lower in cells infected with virus expressing the p14 FAST protein. Expression of p14 FAST protein within cells can cause the fused cells to round up and release from the plate. However, collecting all cells and cell debris into the medium, and centrifuging the sample to collect the material, did not significantly enhance the quantity of tubulin present in the sample. We now show that expression of p14 FAST protein within cells adversely affects the level of certain cytoskeletal proteins within the cell (Figure 7). For example, while infection with CRAd does not significantly alter the levels of vinculin and tubulin within the cells over a 72-h time course, infection with CRAdFAST caused a significant decline in the quantity of these proteins in the cell beginning at 48 hpi. Conversely, the quantity of actin within the cell is relatively unaffected by either virus. p14 FAST protein interacts with N-WASP to induce the formation of a branched actin network that drives cell-cell fusion [87,88]. This actin network provides the mechanical pressure that pushes the membrane-disruptive ectodomain of the p14 FAST protein into adjacent cell membranes, thereby promoting fusion [87]. Thus, actin is important for p14 FAST protein function, likely explaining its apparent stability relative to vinculin and tubulin. The cell rounding frequently observed in p14 FAST protein-expressing cells may be accompanied by a breakdown of the microtubule network, and hence an apparent loss of tubulin stability.

Interestingly, infection with CRAdFAST also caused the formation of a novel, faster migrating species on the histone H3 immunoblot, which was not observed for CRAd (Figure 7). H3 can undergo N-terminal proteolytic cleavage by a number of proteases, termed histone H3 clipping [89]. Although not completely understood, histone clipping may be a mechanism to rapidly remove N-terminal modifications of histones, thus bringing about rapid changes in epigenetic status and gene expression within a cell [90]. For example, cathepsin L was shown to cleave histone H3 at several positions within the N-terminal region in vitro and preferentially between residues A21/T22 during retinoic acid-stimulated differentiation of mouse embryonic stem cells [90]. H3 is also cleaved by granzyme A during straurosporine-induced apoptosis in Raji1 cells [91]. For cells treated with CRAdFAST, expression of p14 FAST protein may destabilize cell membrane integrity, including that of the lysosome, leading to release of lysosome-associated proteases (e.g., cathepsin L), which subsequently leads to the cleavage and degradation of proteins such as histone H3 and tubulin. We have not tested whether other histone proteins are similarly affected. Thus, although additional studies are required to elucidate the mechanism of p14 FAST protein-mediated effects on the stability of different cellular proteins, our data clearly shows that careful consideration must be given to the choice of protein used as a loading control.

## 4. Materials and Methods

### 4.1. Cell Culture

Human 293 [92], 293N3S [93], and A549 lung adenocarcinoma cells [84] were grown in Minimum Essential Medium (Sigma Aldrich, Oakville, ON, Canada) supplemented with 10% (*v*/*v*) Fetal Bovine Serum (FBS) (Sigma Aldrich), 2 mM GlutaMAX (Invitrogen/Gibco, Ottawa, ON, Canada), and 1× antibiotic-antimycotic (Invitrogen). Cells were incubated at 37 °C in a 5% CO_2_ atmosphere.

### 4.2. HAdV and Plasmid Constructs

HAdV used in this study, summarized in Figure 1, are derived from HAdV type 5 and were generated by conventional and RecA-mediated cloning [94]. Oncolytic vectors contain a 24-bp deletion in conserved region 2 (CR2) of the E1A gene, which prevents binding and inactivation of retinoblastoma protein by E1A [95,96]. Our vector CRAd contains the E1AΔ24 mutation and is deleted of the E3 region, and it has been described previously [25]. CRAdFAST encodes the p14 FAST gene with an upstream splice acceptor site derived from the HAdV-40 long fiber transcript (designated herein as 40SA, [53,97]) inserted in place of the E3 region, to allow for replication-dependent expression of p14 FAST from the viral major late promoter (MLP), and it has been described previously [25]. AdRP3089 encodes the monomeric red fluorescent protein (RFP) [98] with an upstream 40SA inserted in place of the E3 region, to allow for replication-dependent expression of the RFP from the viral MLP, as described previously (Ad-late/RFP, [50]).

AdRC129 is an otherwise wildtype HAdV-5 that contains a C-terminal FLAG-epitope tag on ADP. ADP was PCR amplified with synthetic oligonucleotides RC126F 5′-caacgcggccgccgctaccg and RCF126R 5′-gcgctcgaggaatcatgtctcatttaatcacttatcgtcgtcatccttgtaatctccg using pCB6 (a bacterial plasmid containing the entire wildtype HAdV-5 genome flanked by PacI restriction sites) as a template. The resulting product was digested with NotI/XhoI and cloned into NotI/XhoI-digested pBluescript, generating pRC126. This NotI/XhoI fragment corresponds to position 29,511 (NotI) to 29,792 (XhoI) of the conventional HAdV-5 genome, but the PCR amplification introduces the coding sequence for a short linker (glycine-serine-glycine) and a FLAG-tag immediately after the C-terminal amino acid residue of the native ADP protein. This plasmid was sequenced to confirm integrity. The NotI/XhoI fragment from pRC126 was used to replace the analogous region in pCB6ΔBst1107I, placing the FLAG-tagged ADP in the proper context of its downstream (i.e., right-end) sequences of the wildtype HAdV-5 genome, generating pRC127. A 7.6 kb NruI/NotI fragment from pCB6 was cloned into Bst1107I/NotI digested pRC127, adding the native upstream region to ADP-FLAG, generating pRC128. The fragment containing ADP-FLAG and flanking regions was recombined into pCB6, generating pRC129, which was rescued and propagated in 293 cells as virus AdRC129.

AdRC132 is similar to AdRC129 but contains a mutation of the ADP start codon (ATG to TAG, designated mutATG), thus preventing expression of the protein. The ADP region of HAdV-5 (from Bst1107I at position 29,013 to EagI at 29,511) was PCR amplified with synthetic oligonucleotides RC130F 5′atacggccggtatacttttccattttatgaaatgtgcgacattac and RC130R 5′ ataggcggccgcgttggttgtgttggtcTActctgttagggtgggtcgctgtagttg (the mutated nucleotides are shown in capital letters), digested with EagI and cloned into NotI-digested pBluescript, generating pRC130. This plasmid was sequenced to confirm integrity. The 498 bp Bst1107I/NotI fragment from pRC130 was used to replace a similar fragment in pRC128, generating pRC131. pRC131 was recombined into pCB6, replacing the native E3 region with one containing the mutATG-ADP-FLAG, generating pRC132 and the recovered virus AdRC132. Although AdRC132 was not directly utilized in this study, this vector is available upon request.

AdRP3371 contains the E1AΔ24 mutation and the entire E3 region but has the 40SA-p14 FAST expression cassette inserted within, and disrupting, the E3 12.5K open reading frame (ORF) and a C-terminal FLAG-epitope tag on ADP. In this construct, 40SA-p14 FAST is inserted at a BglII site located at position 28,134 of the HAdV-5 genome, which is an identical placement relative to the E3 and MLP promoters as in CRAdFAST. A 2.3 kb BglII fragment, corresponding to one of the two BglII fragments commonly missing from many E3-deleted vectors [99], from pRC129 was cloned into BamHI digested pBluescript, generating pRP3357a. A 2.1 kb MscI/NheI fragment from pRC128 was cloned into MscI/SpeI digested pRP3357a, generating pRP3358, which recreates the contiguous E3 coding region. In parallel, annealed synthetic oligonucleotides 3329F 5′ ctaggaccgcctgaagtctctgattaagcttc and 3329R 5′ ctaggaagcttaatcagagacttcaggcggtc were ligated into XbaI digested pCW101 [24], introducing a HindIII site downstream of the p14 FAST gene, designated pRP3329. A 564 bp HindIII fragment containing the p14 FAST gene from pRP3329 was cloned into HindIII-digested pRP3358, generating pRP3367, essentially adding E3 and additional HAdV-5 homologous sequences downstream of p14 FAST. The fragment from pRP3367 containing 40SA-p14 FAST and the downstream E3 region was used to replace the E3 region of pCW148, generating pRP3371, which was recovered as AdRP3371.

AdRP3372 is similar in structure to AdRP3372 but contains the mutation of the ADP start codon as described for AdRC132. A 2.3 kb BglII fragment from pRC131 was cloned into BamHI digested pBluescript, generating pRP3362a. A 2.1 kb MscI/NheI fragment from pRC131 was cloned into MscI/SpeI-digested pRP3362a, generating pRP3365. A 564 bp HindIII fragment from pRP3329 was cloned into HindIII-digested pRP3365, generating pRP3370. The fragment from pRP3370 containing 40SA-p14 FAST and the downstream E3 region was used to replace the E3 region of pCW148, generating pRP3372, which was recovered as AdRP3372.

AdRC116 encodes the E1Δ24 mutation, along with the p14 FAST and ADP genes separated by the porcine teschovirus-1 self-cleaving 2A peptide sequence (herein referred to as P2A) [74], in place of the E3 region and was constructed as follows. The p14 FAST gene was PCR amplified from pCW102 [25] using oligonucleotides CW100F 5′-ccgccatggggagtggacc-3′ and FAST-R 5′-cacgtctccagcctgcttcagcaggctgaagttagtagctccgcttccagcgtagtctgggacgtcgtatggg-3′, which places an HA-epitope, a Gly-Ser-Gly linker sequence, and two-thirds of the 5-prime sequence coding for the P2A self-cleaving peptide downstream of the p14 FAST gene. A second PCR reaction was performed with oligonucleotides ADP-F 5′-ttcagcctgctgaagcaggctggagacgtggaggagaaccctggacctatgaccaacacaaccaacgcggccgccg-3′ and ADP-R 5′-cggactagtctacttatcgtcgtcatccttgtaatctccgcttcctactgtaagagaaaagaacatgtgtttcagtccg-3′, which adds two-thirds of the 3-prime sequence encoding the P2A sequence onto the 5′ end of the HAdV-5 ADP gene (nucleotides 29,491 to 29,769 of the HAdV-5 genome), and also a FLAG-epitope tag onto the 3′ end of the ADP gene. The resulting DNA fragments were used in an overlap PCR reaction using oligonucleotides CW100F and ADP-R. The sequence of the P2A self-cleaving peptide was (GSGATNFSLLKQAGDVEENPGP) [55,56]. The resulting PCR fragment, encoding p14 FAST-HA/P2A/ADP-FLAG, was digested with NcoI/SpeI and cloned into NcoI/SpeI digested pCW102, generating pRC112, which places the gene downstream of 40SA. The resulting plasmid was sequenced to verify integrity. A 928 bp PvuI fragment from this plasmid was cloned into PacI-digested pDC9 [97], generating pRC114. pRC114 was recombined with pCW148, generating pRC116, an HAdV genomic plasmid containing an E1AΔ24 mutation and the p14 FAST-HA/P2A/ADP-FLAG cassette replacing the E3 region. pRC116 was recovered as AdRC116 virus in 293 cells.

AdRC125 encodes the E1Δ24 mutation, along with p14 FAST-HA ORF and ADP-FLAG ORF separated by an internal ribosome entry site (IRES) derived from the encephalomyocarditis virus (ECMV [57]) replacing the E3 region. The IRES was PCR amplified from pWPI (a gift from Didier Trono (Addgene plasmid # 12254)) and comprises the wildtype sequence of ECMV (NC_001479) from genome position 271 to 833, followed by an engineered ‘ACC’, representing a partial Kozak sequence [100], followed by the start codon used in the native sequence, which was also utilized for our downstream gene. The IRES was PCR amplified using synthetic oligonucleotides 118IRESF 5′gcgactagtctgcaggaattccgcccccccccccctaac and 118IRESR 5′catggtattatcatcgtgtttttcaaag. ADP was PCR amplified with synthetic oligonucleotides 118ADPF 5′ctttgaaaaacacgatgataataccatgaccaacacaaccaacgcggc and ADP-R (described above). The resulting two PCR products were subjected to overlap PCR using oligonucleotides 118IRESF and ADP-R, and the resulting product was digested with SpeI and cloned into SpeI-digested pBluescript, generating plasmid pRC118. pRC118 was sequenced to verify integrity. An 880 bp NgoMIV/SpeI fragment from pCW102 [25] was cloned into NgoMIV/XbaI-digested pRC118, generating pRC120, which places the p14 FAST gene upstream of IRES-ADP. A 1.5 kb XbaI/SalI fragment from pRC120 was cloned into XbaI/SalI-digested pDC5, generating pRC121, which adds the 40SA upstream of the p14 FAST gene. A 1.6 kb PvuI fragment from pRC121 was cloned into PacI-digested pDC9, generating pRC122, placing 40SA-p14 FAST/IRES/ADP between HAdV-5 regions that flank the E3 deletion. pRC122 was recombined into the E3 region of pRP2075 [101], generating pRC123. pRC123 was recombined with pCW130, essentially adding the left end region of the virus (including E1AΔ24), generating pRC125, which was recovered as virus AdRC125.

AdRP3433 contains the wildtype E1 region and the entire E3 region but has the 40SA-RFP expression cassette inserted within the E3 12.5K ORF, similar in structure to AdRP3371. A 1.7 kb XmaI/XhoI fragment from pRP3357a was cloned into XmaI/SalI digested pRP3085 [50], which adds HAdV-5 E3 homology downstream of the RFP gene, generating pRP3385. A 3.5 kb MfeI fragment containing the E3 region from pCB6 was cloned into pRP3385, generating pRP3387. pBHG10 [99] was digested with XbaI/SpeI, recircularized, and designated pRP3398. A 3.8 kb PvuI/AgeI fragment from pRP3387 was cloned into PacI/AgeI-digested pRP3398, adding HAdV-5 homology upstream of RFP, generating pRP3401. A 2.1 kb BsrGI/AgeI fragment from pRP3367 was cloned into BsrGI/AgeI-digested pRP3401, generating pRP3426. pRP3426 was recombined into an HAdV-5 genomic plasmid pRP2468 [50], generating pRP3430. Finally, the wildtype E1 region was introduced into pRP3430 through recombination with pXC1 [26], generating pRP3433, which was recovered as a virus AdRP3433.

AdRP3442 contains the E1AΔ24 mutation and contains the 40SA-p14 FAST followed by a 40SA-ADP cassette replacing the E3 region. An 833 bp EcoRI/SpeI fragment from pRC112 was cloned into EcoRI/XbaI-digested pcDNA3, generating pRC115. pRC115 was digested with BamHI/NotI and annealed synthetic oligonucleotides RC133F 5′gatccgccaccatgaccaacacaaccaacgc and RC133R 5′ggccgcgttggttgtgttggtcatggtggcg were used to introduce a Kozak sequence upstream of the ADP start codon, generating pRC133. A 1 kb SpeI/BclI fragment from pRC133 was cloned into SpeI/BclI-digested pRP2645 [24], generating pRP3284, which was subsequently recombined into the E1/E3-deleted HAdV-5 genomic plasmid pRP2014 and recovered as virus AdRP3287. Thus, AdRP3287 is an E1/E3-deleted virus expressing ADP-FLAG from the cytomegalovirus (CMV) immediate early enhancer/promoter and bovine growth hormone (BGH) polyadenylation signal replacing the E1 region. Although not used in this study, this virus is available on request. A 251 bp BamHI/PflMI fragment from pRC133 was cloned into BamHI/PflMI-digested pRC112 [43], placing ADP-FLAG under regulation by the 40SA and designated pRP3435. A 531 bp XbaI/SpeI fragment from pCW102 was cloned into XbaI-digested pRP3435, generating a tandem cassette of 40SA-p14 FAST and 40SA-ADP, and designated pRP3436. In this construct, the 40SA-ADP gene immediately follows the p14 FAST stop codon, with no additional spacer sequence between the two. A 741 bp SpeI/NheI fragment from pCW105 [25] was cloned into SpeI digested pRP3436, adding HAdV-5 homology downstream of the expression cassette, generating pRP3441. pRP3441 was recombined into pCW148, replacing the existing 40SA-p14 FAST cassette with the tandem 40SA-p14 FAST/40SA-ADP cassette, generating pRP3442, which was recovered as a virus designated AdRP3442.

All HAdV were rescued in 293 cells, and stocks were prepared in 293N3S cells. Viruses were purified by cesium chloride buoyant density gradient centrifugation and titered as described previously [102].

pCI-neo naturally contains an optimized chimeric intron (donor, branchpoint, and acceptor sites) downstream of the CMV enhancer/promoter region [54]. We used this as a base plasmid to investigate alternative splicing between two tandem expression cassettes utilizing the 40SA. pRP3454 was generated by cloning a 916 bp XbaI/SpeI fragment from pRP3436 into pCI-neo. To generate pRP3455, pRP3454 was digested with EcoRI and recircularized. To remove the splice acceptor from the intron naturally contained in pCI-neo, it was digested with BbsI/XhoI, the DNA ends repaired with T4 DNA polymerase, and recircularized, generating pRP3456. A 916 bp XbaI/SpeI fragment from pRP3436 was cloned into XbaI-digested pRP3456, generating pRP3460. To generate pRP3462, pRP3460 was digested with EcoRI and recircularized. These plasmids were tested for expression through transient transfection assay in A549 cells. A549 cells plated in 35 mm dishes were transfected with 2 μg of plasmid per dish using Lipofectamine 2000 (Invitrogen), according to the manufacturer’s instructions. Twenty-four hours post-transfection, the cells were collected in 2 × SDS-PAGE loading buffer (62.5 mM Tris HCl pH 6.8, 25% glycerol, 2% SDS, 0.01% bromophenol blue, 5% β-mercaptoethanol), and analyzed by immunoblot, as described below.

### 4.3. Immunoblot Analysis

A549 cells were seeded into 12-well plates at 0.4 × 10^6^ cells per well and, the next day, were infected with virus at the indicated multiplicity of infection (MOI, defined as viral plaque forming units per cell) for 1 h. Whole-cell lysates were harvested at 24, 48, and 72 hpi in 2 × SDS-PAGE protein-loading buffer, boiled for 5 min, separated by SDS-PAGE, and transferred to polyvinylidene fluoride (PVDF) membrane (MilliporeSigma, IPVH00010, Oakville, ON, Canada). After blocking for 1 h in 5% *w*/*v* non-fat dry milk in Tris-buffered saline containing 0.2% Tween 20 (Thermo Fisher Scientific, Ottawa, ON, Canada), the following antibodies were used to detect the various proteins. Rabbit anti-HAdV-5 polyclonal antibody (pAb) (1:10,000 dilution, Abcam #ab6982, Toronto, ON, Canada) and horseradish peroxidase (HRP)-conjugated goat anti-rabbit IgG secondary antibody (1:10,000 dilution, Bio-RAD #1706515, Mississauga, ON, Canada) were used as a surrogate marker to monitor viral replication. Mouse anti-HA tag monoclonal antibody (mAb) (1:10,000 dilution, Cell signaling #2367) was used to measure expression of the HA epitope-tagged p14 FAST protein. Mouse anti-FLAG mAb (1:1000 dilution, Rockland #200-301-383S, Burlington, ON, Canada) was used to probe for expression of FLAG-tagged ADP. Immunoblot membranes were also probed with rabbit anti-RFP pAb (1:5000 dilution; Abcam, Ab62341), mouse anti-βactin mAb (1:10,000 dilution, MilliporeSigma, A5441) and mouse anti-αtubulin mAb (1:5000 dilution; Calbiochem, CP06, Oakville, ON Canada). The membrane was then washed three times in PBS containing 0.2% Tween (PBST) and incubated with the appropriate secondary antibody conjugated to HRP. Immunoblots were developed using Immobilon Classico Western HRP substrate (MilliporeSigma, WBLUC0500), and visualized by autoradiography.

To more accurately assess protein band intensities, immunoblots were processed for and analysed using an Odyssey CLx imaging system (LI-COR, Lincoln, NE, USA). Separated proteins were transferred to an Immobilon-FL PVDF membrane (Millipore, Oakville, ON, Canada), and the membrane was blocked with Intercept Blocking Buffer (LI-COR). The following primary antibodies and dilutions were used: Rabbit anti-HAdV-5 pAb (1:10,000 dilution, Abcam #ab6982), mouse anti-HA tag mAb (1:10,000 dilution, Cell signaling #2367, Whitby, ON, Canada), mouse anti-FLAG mAb (1:1000 dilution, Rockland #200-301-383S), mouse anti-αtubulin mAb (1/5000 dilution; Calbiochem, CP06), rabbit anti-vinculin mAb (1:10,000 dilution; Abcam, Ab129002) and rabbit anti-Histone H3 mAb (1/15,000 dilution; Cell Signaling, D2B12 #4620S), which were diluted in Intercept Blocking Buffer solution containing 0.2% Tween 20. The membrane was then washed three times in PBST and incubated with the appropriate IRDye secondary antibodies (680RD and 800CW, LI-COR), diluted in Intercept Blocking Buffer solution containing 0.2% Tween 20 and 0.01% SDS, and protected from light. The membrane was washed three times in PBST while still protected from light, followed by a final rinse with PBS. Membranes were then scanned using an Odyssey CLx system and analyzed using Image Studio Lite (version 1.0.9). All protein quantification data are representative of three or more independent experiments.

### 4.4. PCR Analysis of HAdV Transcripts

A549 cells were seeded in 60 mm dishes with 3.2 × 10^6^ cells per plate and infected the following day with CRAdFAST, AdRC129, or AdRP3371 at a MOI of 3 for 1 h. Twenty-four hours later, the medium was removed from the infected cells and total cellular RNA was extracted using TRIzol Reagent (Thermo Fisher Scientific) and the DNA-free removal Kit (Invitrogen-Thermo Fisher Scientific, AM1906), according to the manufacturer’s protocol. An aliquot of the resulting RNA (2 μg) was converted to cDNA using the High-Capacity cDNA Reverse Transcription Kit (Invitrogen-Thermo Fisher Scientific, 4368814) in the presence of RNaseOut Recombinant Ribonuclease Inhibitor (Invitrogen-Thermo Fisher Scientific), according to the manufacturer’s protocol. PCR was performed using a common synthetic oligonucleotide corresponding to a portion of the HAdV-5 L3 region of the tripartite leader, 5′CATCGACCGGATCGGAAAAC, in combination with oligonucleotides hybridizing to fiber (5′GGTTCGGATAGGCGCAAAGA), p14 FAST (5′TAATCCGGCGATCACTTCCC), or ADP (5′GTGTTTCAGTCCGTCCAATCTA). We also examined the total transcript (spliced and unspliced) for each of these genes through the use of primers binding within the open reading frame of fiber (5′TGCGCCTATCCGAACCTCTA and 5′CCTTGGGTGGCAATGCTAAG), p14 FAST (5′TGGGGAGTGGACCCTCTAAT and 5′AGCAAGGCTACTAATCCGGC) and ADP (5′CCGGACTTACATCTACCACAAA) and (5′ GTGTTTCAGTCCGTCCAATCTA). Template (200 ng cDNA) was amplified with Dream Taq DNA Polymerase High Fidelity (Invitrogen-Thermo Fisher Scientific, EP0702), with an initial incubation at 95 °C for 3 min, followed by 25 or 30 cycles of 95 °C for 30 s, 55 °C for 30 s, and 72 °C for 1 min, and a final extension at 72 °C for 10 min. The resulting PCR products were separated by 1.2% agarose gel electrophoresis in TAE buffer (40 mM Tris pH 8.3, 20 mM acetic acid, and 1 mM EDTA) in the presence of ethidium bromide. The major DNA products that appeared in the agarose gel were excised, purified, treated with T4 polynucleotide kinase, cloned into SmaI-digested pBluescript, and sequenced at StemCore Laboratories at the Ottawa Hospital Research Institute.

### 4.5. Immunoprecipitation

A549 cells in 10 cm dishes were infected with HAdV-5, AdRC129, or AdRC125 at an MOI of 100, and lysed 72 h later in modified radio immunoprecipitation assay extraction buffer (50 mM Tris pH 8.0, 100 mM NaCl, 1 mM EDTA, 1% glycerol, 1% NP-40, protease inhibitors). Immunoprecipitation was performed as described previously [103], using 30 μL Protein G beads (1004D, Dynabeads, Invitrogen, Ottawa, ON Canada) for preclearing and 2 μg of antibody (FLAG antibody or IgG (sc-2025, Santa Cruz, Dallas, TX, USA) as negative control). The protein-bound antibody was resuspended in 20 μL 2× protein sample buffer, and the resulting proteins were analyzed by immunoblot, as described above.

### 4.6. Analysis of Virus Spread In Vitro

Plaque assays to assess virus spread were performed as follows. A549 cells were seeded in 6-well plates at 1.0 × 10^6^ cells per well. The next day, the cells were infected with dilutions of HAdV-5, CRAd, CRAdFAST, or AdRC116 for 1 h. The virus was removed by rinsing the plates with phosphate-buffered saline (PBS), and 4 mL of overlay medium was added to each well. Overlay medium was prepared by mixing 3% carboxymethylcellulose (CMC) (*w*/*v*, prepared in water) with equal volume of 2 × MEM containing 20% (*v*/*v*) FBS, 4 mM GlutaMAX, and 2 × antibiotic-antimycotic. At 7 days post-infection (dpi), overlay medium was removed and the cells were fixed with 4% paraformaldehyde (PFA, prepared in PBS) for 30 min and stained with 0.1% crystal violet (*w*/*v*, prepared in water) for 10 min. Plates were then rinsed with cold tap water, inverted and dried overnight, and visualized by microscopy. The relationship between virus dose and virus-mediated cytopathic effect (CPE) was assessed as follows. A549 cells were seeded in 24-well plates at 0.15 × 10^6^ cells per well. The next day, the cells were infected with HAdV-5, CRAd, CRAdFAST, or AdRC116 at an MOI of 0.01–10 for 1 h. The media was removed 7 dpi, and the cells were fixed and stained with crystal violet and analyzed by microscopy. Phase-contrast and bright-field images were captured with a Zeiss Axiovert 200 M microscope (10× objective). Images were processed using ZEN imaging software (3.5 Blue Edition, Zeiss), and the figures were prepared using Inkscape software (version 1.2, The Inkscape Project) or Adobe Photoshop and Illustrator (CS5).

### 4.7. Metabolic Activity Assay

Metabolic activity was quantified using the CellTiter 96^®^ AQueous One Solution Cell Proliferation Assay (Promega, Madison, WI, USA). A549 cells were seeded at 2 × 10^4^ cells per well in flat-bottom 96-well cell culture dishes (Thermo Scientific, Ottawa, ON, Canada). The next day, the cells were either mock infected or infected at an MOI of 10 with CRAd, CRAdFAST, or AdRC116 for 1 h, and fresh medium was applied. Metabolic activity was evaluated at 24, 48, and 72 hpi. In a second approach, A549 cells were infected with varying MOI of the viruses, and metabolic activity was evaluated at 48 hpi. For both experiments, 20 μL of the MTS substrate [3-(4,5-dimethylthiazol-2-yl)-5-(3-carboxymethoxyphenyl)-2-(4-sulfophenyl)-2H-tetrazolium] was added per well and incubated in the dark for 1 h. Color development was determined using a SpectraMax 190 plate spectrophotometer (Molecular Devices, Sunnyvale, CA, USA) at 490 nm.

### 4.8. Statistical Analysis

Statistical analysis was performed using the GraphPad Prism 6 (Version 6.01, GraphPad Software Inc., San Diego, CA, USA). All bar graphs present the mean and standard deviation, unless otherwise indicated. Metabolic activity data presented in bar graphs were compared by two-way ANOVA, and by Tukey’s HSD post-hoc analysis.

## 5. Conclusions

In this study, we have investigated several common methods to achieve co-expression of E3 or ADP within an oncolytic vector armed with a transgene encoding the p14 FAST protein. Use of the P2A self-cleaving peptide in a bicistronic construct was the most efficient at expressing the p14 FAST protein and ADP, but the level of expression achieved for both proteins was reduced relative to our original CRAdFAST vector and wildtype HAdV-5, respectively. Ultimately, reduced transgene expression compromised vector efficacy in cancer cell killing in vitro. Thus, additional studies are required to optimize vector design to achieve high-level expression of multiple therapeutic transgenes in armed oncolytic HAdV vectors.

## Data Availability

The original contributions presented in this study are included in the article. Further inquiries can be directed to the corresponding author.
